# Parental Views on the Psychosocial Impact of False-Positive Results Following Newborn Screening for Severe Combined Immunodeficiency in England

**DOI:** 10.3390/ijns12020026

**Published:** 2026-04-21

**Authors:** Pru Holder, Chloe Musa, Anju Keetharuth, Fiona Ulph, Jim B. Chilcott, Louise Moody, Ellinor K. Olander, Jane Chudleigh

**Affiliations:** 1 Cicely Saunders Institute of Palliative Care, Policy and Rehabilitation, King’s College London, London SE5 9PJ, UK; jane.2.chudleigh@kcl.ac.uk; 2 Division of Psychology & Mental Health, Manchester Centre for Health Psychology, School of Health Sciences, Faculty of Biology, Medicine and Health, University of Manchester, Manchester Academic Health Science Centre, Oxford Road, Manchester M13 9PL, UK; chloe.musa2@nhs.net (C.M.); fiona.ulph@manchester.ac.uk (F.U.); 3 Sheffield Centre for Health and Related Research, University of Sheffield, Sheffield S1 4DA, UK; d.keetharuth@sheffield.ac.uk (A.K.); j.b.chilcott@sheffield.ac.uk (J.B.C.); 4 Centre for Arts, Memory and Communities, Coventry University, Coventry CV1 5FB, UK; aa0445@coventry.ac.uk; 5 Centre for Maternal and Child Health Research, School of Health and Medical Sciences, City St George’s, University of London, London EC1V 0HB, UK; ellinor.olander.1@city.ac.uk

**Keywords:** severe combined immunodeficiency, SCID, newborn screening, false positive, parents, psychosocial, quality of life, mixed methods

## Abstract

The project aimed to explore the psychosocial impact on parents of receiving a false-positive outcome following a positive newborn bloodspot screening (NBS) result for SCID for their child. A mixed-methods design was employed using semi-structured interviews and standardised health-related questionnaires (EQ-5D-5L, ITQOL-47, and GAD-7). The participants were recruited from six National Health Service hospital trusts in England involved in the NHS England In-Service Evaluation of Screening for SCID. A total of 22 interviews were conducted with 28 parents. Health-related questionnaire data were collected from 26 of these parents. The interviews were analysed using a reflexive deductive approach to thematic analysis. For the health-related questionnaire data, a comparison of group means against population norms was undertaken using *t*-tests with unequal variances. The findings from the interviews showed that receiving a false-positive outcome following a positive NBS SCID result could cause parents to have an enhanced view of their child’s vulnerability in the short term. However, negative sequelae were largely mitigated as parents viewed their child’s exposure to ‘normal’ infections as evidence of a functional immune system. The health-related questionnaire data showed that the parents had significantly worse health than the population norm (as indicated by EQ-VAS: *p* = 0.0296); however, all the other measures were non-significant. More research is needed to explore the potential longer-term psychosocial impact of a false-positive screening result for SCID on parents beyond their child’s first year of life.

## 1. Introduction

Severe Combined Immunodeficiency (SCID) is a group of rare autosomal recessively inherited genetic disorders of the immune system characterised by a profound deficiency in cellular and humoral immunity arising from one of many T-cell maturation defects in the bone marrow or thymus gland [1]. Infants with SCID are born asymptomatic, but, within the first few months of life, they can develop severe or recurrent infections, failure to thrive and increased vulnerability to opportunistic infections. The condition can be fatal in the first year of life without early diagnosis and adequate curative treatment, including haematopoietic stem cell transplantation (HSCT), enzyme replacement therapy, or gene therapy [2]. As of June 2024, it was estimated that 42 countries had introduced screening for T-cell deficiency into their newborn bloodspot screening (NBS) programmes [3], which can identify SCID before infections and other signs of SCID develop [4,5,6,7,8]. Data shows improved survival of infants where there is population-based screening for SCID [9].

In 2017 the UK National Screening Committee (UKNSC) recommended an in-service evaluation of the potential introduction of NBS screening for SCID in England [10], henceforth referred to as the SCID evaluation [11]. The SCID evaluation took place between 6 September 2021 and 1 March 2024 across six screening regions, including 13 National Health Service (NHS) trusts in England, involving approximately 800,000 newborns. Screening and data collection are continuing in these areas pending recommendations by the UKNSC. The SCID evaluation utilised the bloodspot already taken by the UK NHS NBS Programme from babies at 5 days old [12] to detect T-lymphocyte receptor excision circles (TRECs). TRECs are a by-product of successful T-lymphocyte receptor formation; low/absent TRECs detected from dried bloodspot samples indicated an increased risk of SCID. Low TREC levels can indicate an increased risk of SCID; however, they may also be influenced by factors such as prematurity or low birth weight, leading to false-positive results. Indeed, during the in-service evaluation, sensitivity and specificity were 100.00% and 99.94%, respectively, but the positive predictive value was only 2.11% [13], reflecting a high number of false positives. This is likely due to the low prevalence of SCID in the screened population, as well as the impact of prematurity on the test results.

Parents are provided with information about NBS during the antenatal period and again at the time the spot sample is taken, which is usually on the fifth day of life. Verbal consent is also obtained from parents before the bloodspot sample is collected. The information provided does not routinely include details about the nature of false-positive results. During the SCID evaluation, following NBS, parents of babies found to be at higher risk of SCID (including both those who had been discharged into the home/community setting and those who were still inpatients in the hospital setting) were notified of the result by phone and an appointment was made for the baby to be seen the next day by an immunology team. At this appointment, the implication of the result was explained by the immunologist/clinical nurse specialist, and another blood sample was obtained to enable flow cytometry to be performed. The flow cytometry results were usually available the same day. A negative flow cytometry result implied a false-positive screening result and the parent and baby could be discharged. In the case of a potentially clinically significant flow cytometry result either appropriate management or further investigation was arranged.

Previous research from other conditions included in NBS programmes has shown that delivering a false-positive result to parents [14,15,16,17,18,19] or asking them to attend a hospital for further tests [8,17,20] following NBS can have both short- and long-term deleterious effects. These effects include elevated parental anxiety, confusion, altered perceptions of child vulnerability and disrupted parent–child interactions, which can persist or become amplified following unclear communication or delayed confirmatory testing [15]. Here we report the views and experiences of parents who participated in the SCID evaluation and whose babies received a false-positive outcome following a positive NBS result for SCID.

The research question addressed was: What are the effects on parents whose baby receives a positive NBS result for SCID (low TRECs) followed by a normal confirmatory test (flow cytometry) (‘false-positive screening result’)?

## 2. Materials and Methods

A mixed-methods design was employed using qualitative semi-structured interviews and standardised health-related questionnaires with parents whose baby received a false-positive outcome following a positive NBS result for SCID in England.

This study was part of a larger programme of work [21] which sought to involve parents whose baby received an SCID, non-SCID TCL or negative result following NBS for SCID, as well as professionals working in the SCID screening evaluation centres. The programme of work was approved by North West—Greater Manchester East Research Ethics Committee (reference: 21/NW/0360).

### 2.1. Setting

Thirteen National Health Service hospital trusts in England that received results from the six NBS laboratories involved in the SCID screening evaluation commissioned by the UKNSC were invited to take part in the study, henceforth referred to as sites. Parents who had received a false-positive outcome following a positive NBS result for SCID were recruited from six of these sites.

### 2.2. Inclusion Criteria

Inclusion criteria can be seen in Table 1. When distributing the recruitment materials, the clinical team were encouraged to make particular effort to ensure the inclusion of people whose first language is not English, Black, Asian, and minority ethnic (BAME) communities and different socio-economic groups (to ensure inclusion of underserved populations), as well as fathers (as male perspectives are under-represented in newborn screening research).

### 2.3. Recruitment and Sampling

Recruitment proceeded via the clinical team. The relevant clinician who gave the family the false-positive outcome following a positive NBS result for SCID provided potential participants with a study “postcard” and/or a Participant Information Sheet (PIS). Parents were given the choice of either contacting the research team directly via contact details listed on the PIS, consenting to share their contact details with the research team, or returning the postcard. Returning the postcard with contact details indicated consent to be sent the relevant PIS with further details of the study. Postage-paid envelopes were supplied and a “request this information in a different language/format” form included with each postcard. Parents who returned a postcard or who agreed to share their contact details with the research team were contacted and asked whether they were happy to be interviewed. Overall, contact details for 70 parents participating in SCID screening were obtained by the research team; 43 of these parents had received a false-positive outcome following a positive NBS result for SCID for their child, of which 28 went on to be interviewed. Written informed consent was obtained from all participants prior to data collection. Parents were made aware of the interview topics and nature of the questionnaires before taking part and were debriefed at the end of each interview to minimise potential distress. Participants were given the name and telephone number of a member of the research team whom they could contact at any time should they have any concerns following their participation.

### 2.4. Development of Data Collection Tools

#### 2.4.1. Interviews

The interviews sought to explore parental experience of NBS for SCID using qualitative methods. The interviews included a technique called ‘journey mapping’. This is a design tool [22,23,24] that can be employed to depict the healthcare service experience from the perspective of the patient or service user and map the ‘touch points’ between the patient and the service. These approaches have been used in previous research to explore and reflect upon parents’ experiences, thoughts, attitudes and emotions throughout the NBS journey [25].

During the interviews, researchers used a set of predefined topics, including the impact of screening on parents, parents’ views of their babies following the screening results, and the care journey. The interview schedules were developed by researchers with expertise in qualitative research in the field of NBS in collaboration with an oversight group consisting of five stakeholders (professionals and patient representatives/parents) through involvement activities. The oversight group met prior to the start of the project to review and provide feedback on the proposed data collection approaches and techniques. The order and precise wording of questions were determined by the interviewees’ responses to elicit more detailed responses.

#### 2.4.2. Questionnaires

Demographic questions were used to describe the sample and were based on those used in previous qualitative studies that have explored families’ experiences of NBS [14,20,26]. They included information such as language spoken, age, ethnicity and family structure. Three validated standardised health-related questionnaires were used to explore and understand health-related quality of life (HRQoL) of the parents and child, parental anxiety and parenting/family burden. These included: the EQ-5D-5L (a generic adult HRQoL measure that comprises five Likert scale questions and one Visual Analogue Scale (VAS) question (EQ-VAS)) [27]; the Infant/Toddler Quality of Life Questionnaire-Short Form 47 (ITQoL-47: an infant QoL measure with 47 questions) [28]; and the Generalised Anxiety Disorder 7-item (GAD-7: a parental anxiety-specific questionnaire with 7 questions) [29]. The EQ-5D-5L comprises five domains—mobility, self-care, usual activities, pain/discomfort, and anxiety/depression—to indicate generic adult HRQoL. The EQ-VAS requires parents to rate their health on a scale of 0–100, with 100 being the best health they can imagine.

### 2.5. Data Collection

Semi-structured interviews with parents were undertaken face-to-face, via telephone, or via Microsoft Teams (depending on parental choice) during the first 24 months after receiving their child’s result (timing decided by parent) between November 2022 and March 2024. Interviews were conducted by researchers with experience in qualitative interviewing (C.M., J.C., and P.H.). Parents were given the option of completing the interviews individually or jointly with their partner. Interviews were audio-recorded and transcribed verbatim. All but one of the interviews were conducted in English without the use of an interpreter service; one interview was conducted with a Latvian interpreter arranged by the research team who translated the data and questions during the interview. Directly after the qualitative interviews, the demographic questionnaire and three standardised health-related questionnaires were administered by the researcher and completed with parents.

### 2.6. Data Analysis

#### 2.6.1. Qualitative Data

Qualitative data from the open-ended questions were transcribed and a deductive codebook thematic analysis was conducted, with the codebook iteratively refined as analysis progressed. Themes were generated using a latent approach involving interpretation of the underlying implicit meanings of the data [30].

After familiarisation with the data, two interview transcripts were coded by six members of the research team (C.M., E.K.O., F.U., J.C., L.M., and P.H.) to aid coding comparisons and inform and align code development [31]. A codebook was developed based on these jointly coded transcripts. A further transcript was then coded separately by the same six members of the research team using the codebook and compared. Following this, the same members of the research team independently coded the remainder of the interviews. All quotes for each theme were collated to inform theme development. This was an ongoing iterative process; new codes were developed and the definition of codes refined as analysis progressed. Once this initial coding had been completed, the final themes were refined.

#### 2.6.2. Quantitative Data

The standardised health-related questionnaires were scored according to their official scoring procedures, except for the EQ-5D-5L, for which the algorithm recommended by the National Institute for Health and Care Excellence was used [32]. Comparison of group means against population norms was undertaken using *t*-tests with unequal variances; these used the reported mean, standard deviation and sample size for the normal population. For the EQ-5D-5L, the norm used was taken from data relating to UK women in the postnatal period [33]. For the GAD-7, the norms used relate to a UK postnatal population [34]. For the ITQOL-47, US norms were taken from the official scoring manual [35]. Because the ITQOL-47 has different norms by age within the time period covered by our analyses, norms were weighted by the ages of participants within each group.

## 3. Results

### 3.1. Demographics

A total of 22 interviews (16 individual and six joint with both mother and father present) were conducted with 28 parents (22 mothers and six fathers) who had received a false-positive outcome following a positive NBS result for SCID, either whilst in hospital (*n* = 11) or at home (*n* = 17). The interview duration ranged from 28 min to 1 h 54 min (median: 54 min). The age of the child when the interviews took place ranged from 2 months to 2 years old (median: 6 months). One parent declined completion of the demographic questionnaire; the demographic characteristics of the 27 parents who completed it can be seen in Table 2.

### 3.2. Themes

Four main themes and five subthemes were identified from the interview data: (i) facing the unexpected: receiving and making sense of a positive NBS result for SCID (the subthemes included (a) information provision, (b) navigating the result at home, and (c) processing the result in the hospital); (ii) life interrupted: emotional response to the false-positive outcome following a positive NBS result for SCID; (iii) what if? Prolonged impact of a false-positive outcome (the subthemes included (a) time moves on, residual concerns and (b) life back to normal); (iv) looking back: would not have it any other way. These are explored in detail below, supported using illustrative quotations from the interview data.

#### 3.2.1. Facing the Unexpected: Receiving and Making Sense of a Positive NBS Result for SCID

##### Information Provision

Parents’ recollection of the provision of pre-screening information was variable, with some having no recollection of receiving information about either screening in its entirety or the individual conditions being screened for specifically. Also, some were not aware of the possibility or likelihood of a positive or false-positive result. Parents reported feeling falsely reassured about the likelihood of receiving a negative NBS result, which further compounded reported feelings of shock and fear when they received the initial positive NBS result for SCID.

*“Basically, you know, you spend the night going, ‘We’ve got to go to hospital tomorrow….’ And that’s really scary, and there’s no getting around it. It’s horrible, you know.”*

*Father (P1)*

Being provided with information after receiving the initial positive result helped to alleviate some of the parents’ concerns, although which parents benefited most from this form of information provision (i.e., according to demographic categories) was not discernible from the data.

*“…she was really reassuring…she gave me plenty of information. She sent everything through to my email, she checked that I’d received them as well, so while I was on the phone she got me to check that I’d got everything—which I had. …But yeah, all the information was great. She did give me an overview of what SCID was—not too much, didn’t bombard you—but yeah, she made me understand, as well, the seriousness of it.”*

*Mother (P34)*

Despite being provided with information and being advised not to seek further information online during the time frame between receiving the initial result and confirmatory testing, most parents searched for additional information via the internet. However, this was often unhelpful and frequently increased anxieties parents were already experiencing.

*“…you go on and google, you get all the worst case information, and I think that was the scary part because it tells you all the worst things.”*

*Mother (P26)*

##### Navigating the Result at Home

For families whose babies were in the community at the time of notification of the positive NBS result, communication occurred via the telephone. This could pose problems as it was often unexpected, from a private/withheld number, and, for some parents, this occurred while in a location/position that was not amenable to answering the call. In addition, information about the initial positive NBS result was frequently communicated to one parent while they were on their own. This meant the onus was placed on them to relay this information to their partner/significant other.

*“You don’t get much like advanced warning, that throws you a little bit and my husband wasn’t at home… you get on the phone and you’re trying to relay the information… it’s obviously easier for you both to be able to digest it when you’ve both had it first-hand.”*

*Mother (P12)*

For some parents, being called by a specialist team and sometimes by a consultant immunologist highlighted the potential seriousness of the NBS outcome. Meanwhile, others reported being reassured as they were able to speak directly to a specialist and have any immediate questions they had answered appropriately. Again, addressing the possibility of the NBS result being a false-positive also helped to allay parental fears and anxiety while awaiting confirmatory testing.

*“…he’s an expert and extremely well-versed but pitched it on a level for us where he broke the harsh news…explained…the fact that SCID was very rare…and he said, ‘Look, if he’s fine and thriving and he’s okay, I think the chance of him having SCID are very, very small. It’s a very rare disease but I can’t ignore it, and the only reason I’m getting you to come to [hospital] is because our lab’s here so we can get the blood straight to those labs.”*

*Father (P1)*

During communication of the initial positive NBS result for SCID, parents were advised that they would need to bring their child to the hospital, normally the next day, for confirmatory testing. However, this could pose practical problems in terms of juggling having a new baby and, for some parents, older siblings. It also required consideration of practicalities and financial resources needed to attend a hospital appointment at such short notice.

*“…who’s going to look after my little girl [older sibling], who’s going down to the hospital, we can’t both be down there again, because she needs a parent.”*

*Mother (P62)*

*“…we don’t have a car to transport the child down there, and the child was still new, so I was wondering how I would move around. So, my friend just said, We’ll get on the bus,’ because taking a taxi down there would be quite expensive, because we checked…then I checked the price, £20 to £25. The same thing when you come back. So, I’m spending, like, £40 to £50, to and from. I don’t know. That was quite expensive.”*

*Mother (P5)*

While the urgency with which children were seen at a specialist centre could be logistically difficult, parents were appreciative of the lack of delay between communication of the initial positive NBS result and confirmatory testing. Despite this, parents still reported issues with sleep, emotional distress and denial during this interim period of uncertainty.

*“I think that was-, it was the shock, I think, with the SCID thing and it was that we were just left to, sort of, fester overnight… that evening, that night, we didn’t sleep…me and my husband took it in turns to have panic attacks. It was just awful. Yes, it was honestly one of the worst nights of our lives because we just thought, well, we’re on a knife edge here. We could have this awful disease…when we put the phone down at quarter to five, we were left in our own heads until 9:30 the next morning and it wasn’t until, say, two o’clock the next day that we got the results.”*

*Mother (P67)*

##### Processing the Result in the Hospital

Distress associated with being informed of their child’s positive NBS result was exacerbated for parents of babies who were inpatients in the hospital setting (e.g., in the neonatal intensive care unit). The reasons for this were multifaceted and linked to potentially already traumatic and stress-inducing experiences, such as the premature birth of the baby, detection of unexpected co-morbidities in the baby and lack of access to support networks during this time.

*“I was on my own…very scary, because you’re miles away from home really [admitted to neonatal unit due to extreme prematurity] and you’ve got nobody to talk to and, you know, share your concerns and your worries with and that’s really hard, because she was very ill anyway, so touch and go at the time. So, to have that on top was worrying…shock, I think, because we felt, you know, really unlucky because, ‘How can she have that when she’s already fighting with what she’s got?’”*

*Mother (P7)*

Parents whose baby was an inpatient at the time of screening suggested that greater consideration should be given to how communication of the positive NBS for SCID is managed. This was due to the numerous invasive procedures their baby was already undergoing, and therefore the quantity of information they were having to process, in most cases unexpectedly. It was proposed that parents could be given the choice at the time of screening to opt not to be informed if the screening result was positive and only be told if the follow-up confirmatory tests identified anything of clinical significance. One family experienced this: their child was an inpatient in the neonatal unit and they were only provided with minimal information about the positive NBS result and in hindsight appreciated how this had minimised the impact of their experience.

*“I was happy to not know much about it…I’d have maybe gone home panicking about it, but we at least had a nice week of blissful ignorance…I didn’t worry. I don’t think because they said very much, it was probably a false positive because he’s pre-term. Had they then given me lots of information booklets about SCID, I might have gone home worrying. And if I didn’t need to worry, I’m glad I could have a nice week with him and introduce him to my mum and dad, and your mum and dad, without thinking, ‘He’s probably got this horrible genetic condition that we’re not going to find out about for another week or two.’”*

*Mother (P10)*

However, another parent expressed the importance of being kept informed and involved in any decision-making about their child.

*“I think one of the things that I found really important, even now, is that even though I didn’t have a definite answer, I was still kept in the loop before they knew for sure… I didn’t feel like anything was being hidden, information wise, that’s a really important point as well. So when we were told, you know, she could have it, I was really grateful to be kept informed like that.”*

*Mother (P7)*

Parental preference for how much information was provided in the inpatient setting varied at the individual level, both across demographic groups and in some cases within couples.

#### 3.2.2. Life Interrupted: Emotional Response to the False-Positive Outcome Following a Positive NBS Result for SCID

For most parents, a false-positive confirmatory result was met with an immense sense of relief and gratitude that their child had been screened despite the additional anxiety the process had caused.

*“Just so relieved…you feel like you could collapse ’cause you’re just so relieved. You know, you think your life could be completely different, to then hear it’s going to be alright and everything’s gone back to normal… just so relieved, so happy…just go home and pick up from where you left off.”*

*Mother (P34)*

However, for other parents, the false-positive confirmatory result left them with a sense of confusion or resentment that they had gone through a stressful experience and unnecessary additional tests with no clinical benefit for their child. This feeling was more common among parents who were notified of the result in the inpatient setting, perhaps due to the potentially already traumatic and stress-inducing events they experienced.

*“…my husband burst into tears because it was just, like, relief, I think. I felt really angry that we’d been put through this awful period. I felt that we’d been through a trauma that had come out of the blue, like, we weren’t expecting it and then it was just, we were sent on our merry way, kind of thing.”*

*Mother (P67)*

These parents commonly spoke about how the experience had negatively impacted their mental health. Many parents likened this to post-traumatic stress disorder and talked about triggers they experienced that contributed to their ongoing anxiety and distress. This was exacerbated by a feeling of abandonment and lack of follow-up support offered after receiving the false-positive confirmatory result. Some parents discussed using prescription medications to treat their ongoing symptoms of anxiety and depression.

*“…there were occasions I think where, you know, I motor on and I’d be fine, a couple of months after [baby] was discharged, and then there would be something triggering like a triggering piece of music we’d heard or something, and I would just burst into tears…follow up would have been good. Just because I think, obviously the false positives, they’re not seen as important”*

*Mother (P14)*

#### 3.2.3. What if? Prolonged Impact of Screening

##### Time Moves on, Residual Concerns

There were mixed feelings between parents relating to their child’s health following the NBS result, with no clear pattern emerging in terms of which parents experienced a prolonged impact compared with others. Some parents described residual concerns, which meant they took additional steps to isolate their child as they feared they may contract an infection that they would not be able to fight.

*“We’d never to take her to soft play or, like, baby groups while she’s this young. I think we’re just too scared… In case she gets an illness or something, and she never bounces back from it. I think it is due to her condition as well, but obviously, the SCIDs-, we always say, ‘Imagine she had that SCIDs. Like, what would we do?’… I’d never take her to a shop on, like, Saturday afternoon. I would only go of, like, a night-time if it was a bit quieter.”*

*Mother (P4)*

Some parents still demonstrated the need to rationalise their decision-making.

*“I even questioned recently, even though I know there’s a negative result and he’s just doing really well now, he’s home and he’s fine… I have mentioned that, ‘Do we need to do a follow-up? Do we need to do another blood test now? Should things be checked because we had this in the past and it was a positive, then it was a negative?’ …I’d like to draw a line under everything that happened… I just go through things over the time and go, ‘Actually, we should have probably had a follow-up. We should have maybe had a little chat about all that went on.’ But it’s not like that.”*

*Mother (P35)*

*“…But we do have to reassure ourselves all the time and we’re, like, ‘Look, she’s been fully checked by the top people.’”*

*Mother (P67)*

In addition, the impact on parents’ mental health had also decreased but was not always resolved.

*“I’m a bit more weary now when things are done to [baby]… I ask every question under the sun… it’s easing over time, when she was a newborn I wouldn’t take her out of the house. I wouldn’t even take her up the road for a walk, I’d be too paranoid, but it is getting a lot better now, like, my auntie does come round and she sits with her more…”*

*Mother (P4)*

##### Life Back to Normal

Some parents described how life following NBS had returned to normal as they were reassured by the fact that their child had had numerous infections and remained well, which they understood to mean that their child did not have SCID. For many parents, the experience had given them an increased appreciation of their child’s good health.

*“He’s in great health, like, I’d put him in the same as me probably…his immunity is really good. Yes, he goes to nursery four days a week and a couple of occasions there’s been, like, a cold that’s wiped people and he still keeps going because he seems to be fine with it. Yes, he seems really happy and healthy, to be honest.”*

*Mother (P14)*

Most parents felt that, as time went on, they thought about the SCID NBS experience less and less.

*“It’s a pretty instant relief. I don’t have any residual, bad feelings about it, like it was a horrible, well twenty-four hours, it was scary…but then you know it was huge relief and it was all okay I think after that.”*

*Mother (P10)*

#### 3.2.4. Looking Back: Would Not Have It Any Other Way

Despite the positive NBS result leading to distress for some parents, all the parents were pleased that their child had been screened and were in favour of NBS for SCID being added to the national NBS programme.

*“I’m glad we did it and it wasn’t down the line when [baby] was a bit older and suddenly symptoms start, or things like that, and then they’re, like, ‘Oh, he’s got this now.’ …I think getting it when they’re younger, then you have more of a chance to find out the best routes and do what you can…I think ultimately the quicker you find this information out the better down the long term. So, I think it should be done.”*

*Mother (P35)*

In hindsight, parents also expressed a view that the importance of NBS for SCID outweighed the associated stress and anxiety of a false-positive confirmatory test.

*“…although it was stressful and we, you know, were worried and really anxious about it, I wouldn’t have changed it, I wouldn’t have had it any other way. Because I think it’s really important because if he would have had SCID and, you know, this trial didn’t exist and, you know, it wouldn’t have been picked up.”*

*Mother (P38)*

### 3.3. Health-Related Questionnaires

Standardised health-related questionnaires were completed with 26 parents (EQ-5D-5L, EQ-VAS, and GAD-7 results shown in Table 3). The mean EQ-5D-5L scores for the study population were below the population norms (indicating worse parental health compared with the general population), although this difference was not statistically significant at the 5% level (*p* > 0.05). The EQ-VAS scores were significantly lower than the population norms (*p* = 0.03), indicating worse health compared with the general population). For the GAD-7, the parental anxiety levels were higher than the population norms (indicating higher levels of anxiety); however, this difference was not statistically significant at the 5% level (*p* > 0.05).

The ITQOL-47 produces nine domain scores (listed in Table 4), although one of these (combined behaviour) cannot be generated for children under the age of 12 months. There was good evidence of reduced HRQoL within the first year for the parents and infants compared to the population norms (Table 4). There was also strong evidence of reduced general health perceptions at one year post birth compared with the population norms (*p* < 0.01). This domain is based on six questions relating to the child’s overall health, how the parent feels their child’s health compares with other children, whether their child seems to pick up infections more frequently, whether they expect the child to have a healthy life and whether they worry about their child’s health more than other parents. There was also strong evidence of lower parental emotional impact scores compared with the population norms (*p* < 0.05). This domain is based on four questions concerned with the amount of parental worry relating to their child’s physical health, emotional health, learning/cognitive abilities and interactions with others. The differences within all the other domains were not statistically significant at the 5% level (*p* > 0.05).

## 4. Discussion

The data presented are part of a larger study exploring parental experiences of screening for SCID within the NHS England SCID Evaluation. These data focus on the immediate and potential longer-term sequelae experienced by parents following the initial positive NBS result and upon receiving a false-positive confirmatory result for SCID.

Depending on the stage of their NBS journey, the emotions experienced by parents in response to their child’s false-positive NBS SCID result ranged between shock, fear, relief and anger, highlighting the importance of recognising NBS as a journey rather than simply the point at which the NBS sample is taken [36,37]. For some families, uncertainty remained after the confirmatory test results. There was also evidence of reduced HRQoL within the first year for parental perceptions of their child’s health compared to population norms using the ITQOL. Despite this, all the families were very much in favour of the introduction of screening for SCID and expressed altruistic views about the importance of recognising and treating children with true SCID early even if this meant some families experienced what appeared to be mainly transient anxiety associated with a false-positive SCID result.

All the parents reported a range of negative emotions associated with receiving the initial positive NBS result and reported the period of waiting time between the initial NBS result and repeat testing/confirmatory result as being particularly stressful. A study in the UK with mothers following a false-positive NBS result for cystic fibrosis (CF) similarly found that nearly two thirds recalled that the wait for the results of repeat tests was difficult and led to emotions such as upset, guilt and increased anxiety [38]. Similar experiences have been identified in a range of other conditions, such as congenital hypothyroidism and Krabbe disease [15,20,39,40]. Minimising the time between communication of the positive NBS result and confirmatory diagnostic testing [38,41] has shown to be an important measure to reduce potential anxiety in parents while awaiting the outcome of NBS. Parents in the present study were appreciative of the short time they had to wait in between communication of the initial positive NBS result for SCID and confirmatory testing even though this highlighted the potential seriousness of the condition.

Parents in the present study felt that negative emotions experienced whilst waiting for the confirmatory result could have been mitigated if there had been appropriate information provision, including a discussion with a key healthcare professional about the possibility of a false-positive result at the time the ‘heel prick’ sample was taken and upon receiving the initial NBS result. This fits with previous research, which highlights the importance of preparing parents for the possibility of being recalled for further tests [20,37], and would seem particularly important for SCID NBS given the relatively high number of false-positive results detected. However, previous research has also shown that it can be difficult for parents to understand condition-related NBS information [42], and the way in which information is provided can be as important as the content itself [43]. This is particularly relevant given recent developments in genomic screening as the challenges surrounding information provision and informed consent are likely to be exacerbated by the increasing number of conditions that may be included in future NBS programmes [44].

In addition, for babies who are inpatients in the neonatal intensive care unit at the time of the ‘heel prick’ test, there is a greater likelihood of them receiving a false-positive outcome following a positive NBS result for SCID [45]. As such, it may be beneficial to consider giving parents of these babies the choice of being informed of the initial positive NBS result for SCID or waiting for the results of the confirmatory testing to reduce their burden of uncertainty at what is already an extremely stressful and emotive time. Permission would be sought at the time of the bloodspot sample being taken to delay reporting results until confirmatory testing is available given the high rate of false-positive results seen in premature babies. This would also provide an opportunity to discuss the possibility of a false-positive result, which is not currently addressed. Parents of babies who were inpatients in the hospital setting in the present study described exacerbated distress, often due to the cumulative effect of a false-positive result for SCID along with other medical issues. Optimising the way parents receive information about NBS and all the potential outcomes antenatally, as well as informing them about the likelihood of a false-positive result if these data are available (as well as the provision of practical information and resources to attend the hospital for parents in the community), would therefore be beneficial [20,37]. This should include links to trusted websites should families wish to learn more about the process or the condition [26,40,46]. This is important since, despite parents frequently being advised not to use the internet to search for information about their child’s NBS result during the time between the communication of the initial NBS result and the confirmatory diagnostic testing, studies have found that almost all parents do this, with potentially harmful effects to their mental health [14,20,26,47]. Previous research has also shown that the provision of more information from a variety of sources is possibly unlikely to be effective in informing parents about NBS as parents generally limit NBS information-seeking to one source [43]. This would suggest that strategies to optimise the way information about SCID NBS is delivered to parents, to enable maximum understanding and appreciation, needs careful consideration.

Parents reported mixed experiences about whether the negative emotions associated with an initial positive NBS result subsided over time or not following a confirmed false-positive result. Some parents continued to feel worried and cautious about their child’s health, which led them to be careful and adopt extra precautions to prevent their child from being exposed to what they considered to be unnecessary risks. This was, for some parents, exacerbated by a lack of after-care following the confirmed false-positive result. A recent review found that abnormal NBS results, even when repeat testing was normal (i.e., a false-positive result), were associated with parental anxiety and/or depression [15].

Whilst for some parents the negative emotions associated with an initial positive NBS result persisted, for others they subsided over time. Viral infections are common in children, especially in the first few years of life, but are normally self-limiting [48]. However, for children with SCID, these normally self-limiting infections can be very serious and potentially life threatening [49]. Therefore, for parents who had received a false-positive result for SCID, the fact that their child had experienced numerous infections and remained well during their first year of life acted as reassurance and additional confirmation that their child did not have SCID. Indeed, for many parents, the experience provided them increased appreciation of their child’s good health. A Canadian study explored the impact of false-positive NBS for CF using validated scales to measure anxiety, distress, maternal perception of child vulnerability and perceived uncertainty related to childhood illness. The findings indicated that, two months after the child’s birth and one year later, the mean anxiety and distress scores were low and did not differ from a control group [41].

Strengths and limitations: The design, data collection, and analysis strategy for this study were influenced by members of a stakeholder oversight group, which included parents of children who had taken part in the NBS programme. The participants were recruited from six geographical locations in the UK and included a range of mothers and fathers, which increases the transferability of the findings. However, we recognise that, due to the difficulties in recruiting this population, we were unable to recruit a large enough sample to meaningfully compare findings across different demographic groups. For example, the number of parents who received the result whilst their baby was in the neonatal intensive care unit is relatively small (*n* = 11) considering that these babies made up a larger proportion of those receiving a false-positive result for SCID. The EQ-5D-5L and EQ-VAS questionnaires are generally self-reported, and we recognise that the presence of an interviewer during their completion could have potentially impacted the results. Furthermore, whilst the findings of this paper focus on data from initial interviews with parents, follow-up data collection with parents is ongoing (annual interviews until the child’s fifth birthday), which will enable the assessment of the longer-term impact. Finally, whilst the scope of this paper was to consider false-positive results, future research may consider the prevalence of false-negative SCID screening cases and their potential impact on parents, although no false-negative cases were detected during the in-service evaluation [13]. For example, a previous study showed that the diagnostic process for CF is worse after a false-negative screening result, leading to negative impacts on parental employment, childcare arrangements and parent/child relationships [50].

Recommendations for practice: The findings from this study suggest a number of recommendations for practice. For example, parents should be advised of the potential outcomes, including the possibility of a false-positive result, where this does not already happen. Strategies to optimise the way information about SCID NBS is delivered to parents to enable maximum understanding and appreciation require further consideration. Parents of babies who are inpatients (e.g., in the neonatal intensive care unit) should be given the option, when they consent to NBS, of only receiving the result of confirmatory testing due to the increased chance of a false-positive result. Clinical teams should consider offering parents who have received a false-positive result for SCID a follow-up appointment at one year to address any residual concerns, refer parents to perinatal mental health services and ensure the health visiting teams are informed so they can provide appropriate follow-up and support for parents.

## 5. Conclusions

The communication of a positive NBS result for SCID was distressing for parents, but revisiting the way pre-screening information is provided to parents, and therefore the informed consent process, may help to mitigate this for the whole of the NBS programme. A false-positive outcome following a positive NBS result for SCID could cause parents to have an enhanced view of their child’s vulnerability in the short term, but, due to the nature of SCID, exposing children to ‘normal’ infections in the first year of life provided evidence to parents that their child’s immune system was functional, and in many cases this helped to mitigate the long-term negative sequelae associated with this.

## Figures and Tables

**Table 1:** Inclusion criteria.

Inclusion Criteria
Parents whose baby received a false-positive SCID screening test result (i.e., received a positive NBS result for SCID, were given an appointment with an immunologist, and received a normal result following confirmatory testing).
At least 18 years of age. Able to understand the purpose and implications of the research study.

**Table 2:** Demographic characteristics.

Demographic Characteristics	Total (*n* = 27)
Parental Age	
18–25	1
26–30	9
31–35	9
36–40	4
41–45	3
Not answered	1
Marital Status	
Married/in partnership with co-parent	25
Separated from co-parent	2
Ethnicity	
English/Welsh/Scottish/Northern Irish/British	21
Any other White background	3
African	1
Any other Asian background	1
Indian	1
First Language Spoken	
English	23
German	1
Latvian	1
Polish	1
Punjabi	1
Religious Background	
No religion	16
Christian	9
Jewish	1
Sikh	1
Level of Qualification Achieved	
A qualification at degree-level or above	19
AS, A-Level equivalent	3
An apprenticeship	2
GCSEs or equivalent	2
Other qualifications	1
Employment Status	
Employed full-time	12
On maternity/paternity leave	8
Employed part-time	4
Looking after home/family	2
Carer for a disabled child	1

**Table 3:** EQ-5D-5L, EQ-VAS, and GAD7 scores compared to population norms.

Parental Instrument	Sample Mean, (SD), [*n*]	Population Norm Mean, (SD), [*n*]	Mean Difference (Observed Minus Norm)	*p*-Value
EQ-5D-5L ^1^	0.81 (0.20) [19]	0.86 (0.18) [493]	−0.05	0.318
EQ-VAS ^1^	79 * (15) [20]	87 (14) [423]	−8.00	0.030
GAD-7 ^2^	4.79 (4.66) [19]	3.20 (3.96) [283]	1.59	0.163

^1^ Higher values represent better health. ^2^ Higher values represent greater anxiety. * Statistically significant (*p* < 0.05).

**Table 4:** Mean ITQOL domain scores compared to population norms.

ITQOl-47 Domain	Sample Mean, (SD), [*n* = 26]	Population Norm Mean, (SD), [*n* = 493]	Difference (Observed Minus Norm)	*p*-Value
Physical abilities ^1^	90.39 (22.80)	96.06 (15.88)	5.67	0.241
Growth and development	89.62 (18.77)	96.02 (11.57)	6.40	0.080
Pain	74.52 (20.46)	76.7 (18.23)	2.18	0.599
Temperament and moods	80.29 (12.05)	83.25 (12.30)	2.96	0.233
Combined behaviour ^2^	79.58 (13.33)	79.42 (12.04)	−0.16	0.977
General health perceptions	64.10 ** (19.29)	81.21 (13.26)	17.11	<0.001
Parental emotional impact	82.21 * (18.26)	92.02 (15.56)	9.81	0.012
Parental time spent	94.23 (12.20)	91.41 (19.06)	−2.82	0.275
Family cohesion	87.12 (18.50)	81.69 (20.62)	−5.43	0.158

^1^ The ‘physical abilities’ domain score could not be calculated for two infants due to missing data, leaving *n* = 24. ^2^ Scores for the ‘combined behaviour‘ domain do not exist for very young infants; consequently, only six observations (*n* = 6) are available, and *n* = 246 are available for the sample population and normal population, respectively. * Statistically significant (*p* < 0.05). ** Statistically significant (*p* < 0.01).

## Data Availability

The data are not publicly available due to ethical constraints.

## Abbreviations

The following abbreviations are used in this manuscript:

BAME	Black, Asian, and Minority Ethnic
CF	Cystic Fibrosis
EQ-VAS	EuroQol-Visual Analogue Scale
GAD-7	Generalised Anxiety Disorder 7-Item
HRQoL	Health-Related Quality of Life
HSCT	Haematopoietic Stem Cell Transplantation
ITQOL-47	Infant/Toddler Quality of Life Questionnaire-Short Form 47
NBS	Newborn Screening
NHS	National Health Service
PIS	Participant Information Sheet
QoL	Quality of Life
SCID	Severe Combined Immunodeficiency
TRECs	T-Lymphocyte Receptor Excision Circles
UKNSC	UK National Screening Committee
